# Supplementary material for: An Evaluation of Antibiotic Prescribing Practices in a Rural Refugee Settlement District in Uganda
Source: Antibiotics (Basel). 2021 Feb 9;10(2):172. doi: 10.3390/antibiotics10020172 (PMC7915286; doi:10.3390/antibiotics10020172)
Supplement: Supplementary file 1 [file antibiotics-10-00172-s001.pdf]

antibiotics

MDPI

Article

An evaluation of antibiotic prescribing practices in a rural refugee settlement District in Uganda.

Matua Boniface [1,3], Winnie Nambatya [2] and Kalidi Rajab [2,3,*]

Supplementary file

Table S1 showing percentage of encounters with one or more antimicrobial medicines prescribed

Health facility Number	No. of prescriptions in the review period	Total of prescriptions with antimicrobials prescribed	% of encounters with antimicrobials prescribed	WHO standard
1	7,494	1,950	26%	20-26%
2	7,710	1,873	24%	
3	7,232	1,696	23%	
4	10,794	2,797	26%	
5	11,930	2,086	17%	
Total/Average	45,160	10,402	23%	

Table S2 showing percentage of antimicrobial medicines prescribed by generic name

Health facility Number	No. of medicines prescribed	No. of medicines prescribed by generic name	% medicines prescribed by generic name	WHO standard
1	115	68	59%	
2	121	78	64%	100%
3	146	74	51%	
4	129	71	55%	
5	158	78	49%	
Total/Average	669	369	55.2%	

Table S3 showing average number of antimicrobial medicines prescribed per patient

Health facility	Patient number	Single prescriptions	Two or more Prescriptions	Total antimicrobial medicines prescribed	Prescription per patient	WHO standard
1	100	85	15	115	1.15	≤ 2 (1.6-1.8)
2	100	83	17	121	1.21	
3	100	62	38	146	1.46	

4	100	72	28	129	1.29
5	100	54	46	158	1.58
Average	100	71.2	28.8	133.8	1.338

Table S4 showing percentage encounters with Antimicrobial injection prescribed

Health facility	Patient number	No of patients given an injectable	% of encounters with an injection prescribed
1	100	20	20%
2	100	19	19%
3	100	27	27%
4	100	22	22%
5	100	37	37%
Total	500	125	25%

Table S5 showing average cost of antimicrobials prescribed per patient

Item	HEALTH FACILITIES					
	1	2	3	4	5	Total
Total cost of essential Medicines as per current invoice (UGX)	3,678,543	3,712,775	3,695,673	11,276,295	9,310,369	31,673,655
Total cost of antimicrobial medicines as per current invoice (UGX)	2,061,480	1,877,980	1,618,580	3,096,310	3,250,520	11,904,870
Percentage cost contribution of antimicrobials	56%	51%	44%	27%	35%	38%
Average cost of antimicrobials per prescription (UGX)	1,592	1,581	1,560	2,132	3,011	1,975

Table S6 showing average duration of prescribed antimicrobial treatment

No of days	Health facilities					
	1	2	3	4	5	Total	Percentage (%)
3	17	14	9	2	9	51	10
5	83	86	88	92	91	440	88
7	0	0	3	6	0	9	2

Table S7 showing availability of a set of key antimicrobials in the facility stores on the day of the study

No.	Name of medicine	Available on the day of visit (yes=1, no=0)					
		1	2	3	4	5	Total
1.	Amoxicillin 250mg capsule	1	0	0	0	1	2
2.	Amoxicillin 250mg dispersible tab	1	1	0	1	1	4
3.	Ampicillin 250mg/cloxacillin 250mg inject.	0	0	1	1	1	3
4.	Ceftriaxone sodium 1g powder for inject	1	1	0	0	1	3
5.	Chloramphenicol sod. Succi. 1g injection	0	1	1	1	0	3
6.	Ciprofloxacin 500mg tablet	1	1	0	1	1	4
7.	Cloxacillin 500mg im (iv)	0	1	0	0	0	1
8.	Cotrimoxazole 960 mg tablet	1	1	1	1	0	4
9.	Doxycycline 100mg capsules	1	1	1	1	0	4
10.	Erythromycin 250mg Tab	1	0	0	0	1	2
11.	Gentamycin 80mg/2ml injection iv/im	1	0	0	1	1	3
12.	Metronidazole 200mg	1	1	0	1	1	4
13.	Nitrofurantoin 100mg tablet	1	1	0	1	1	4
14.	Penicillin, Benzathine benzyl 2.4mu/1	1	1	1	1	1	5
15.	Penicillin, procaine 3mu+ benzyl 1mu	1	1	1	1	1	5
	Total	12	11	6	11	11	51
	Average	80%	73%	40%	73%	73%	68%
	Average HCIII						64%
	Average HCIV						73%

Table S8 showing average number of days that a set of key antimicrobials is out of stock

No.	Name of medicine	No. of days out of stock in the past 3 months (March-May)					
		1	2	3	4	5	Total
1.	Amoxicillin 250mg capsule	0	7	31	16	0	54
2.	Amoxicillin 250mg dispersible tab	12	0	31	0	0	43
3.	Ampicillin 250mg/cloxacillin 250mg inject.	47	21	0	0	0	68
4.	Ceftriaxone sodium 1g powder for inject	0	5	17	25	0	47
5.	Chloramphenicol sod. Succi. 1g injection	47	0	0	0	31	78
6.	Ciprofloxacin 500mg tablet	0	0	19	0	0	19
7.	Cloxacillin 500mg im (iv)	47	0	31	23	31	132
8.	Cotrimoxazole 960 mg tablet	0	0	0	0	31	31
9.	Doxycycline 100mg capsules	0	0	0	0	8	8
10.	Erythromycin 250mg Tab	0	17	0	8	0	25
11.	Gentamycin 80mg/2ml injection iv/im	0	28	31	0	0	59
12.	Metronidazole 200mg	0	0	31	0	0	31
13.	Nitrofurantoin 100mg tablet	0	0	31	0	0	31
14.	Penicillin, Benzathine benzyl 2.4mu/1	0	0	0	0	0	0
15.	Penicillin, procaine 3mu+ benzyl 1mu	0	0	0	0	0	0
	Total	153	78	222	72	101	626
	Average	10.2	5.2	14.8	4.8	6.7	8.3
	Average HCIII						10
	Average HCIV						5.8
